# Synthesis of Er^3+^:YAG Nanocrystals and Comparative Spectroscopic Analysis with Bulk Counterparts

**DOI:** 10.3390/mi14020255

**Published:** 2023-01-19

**Authors:** Chris Rightsell, David Sanchez, José Escudero, Eduardo Ortega, Gangadharan Ajithkumar, Dhiraj Sardar, Arturo Ponce

**Affiliations:** 1 Department of Physics and Astronomy, The University of Texas at San Antonio, San Antonio, TX 78249, USA; 2 Department of Science and Mathematics, Texas A&M University San Antonio, San Antonio, TX 78224, USA

**Keywords:** rare-earth nanoparticles, YAG, laser materials

## Abstract

Single-crystal Er^3+^:YAG has long been used as a laser material, and recent work has shown polycrystalline ceramic Er^3+^:YAG to be a suitable laser material, with benefits of lower cost and easier production. However, relatively little work has been done with the synthesis and spectroscopic characterization of Er^3+^:YAG nanocrystals. In this work, we present the synthesis of nanocrystalline Er^3+^:YAG and the results of comparative spectroscopic characterization with single-crystal and polycrystalline ceramic counterparts. The results show good agreement between the optical properties of the three hosts, with the nanocrystals demonstrating relatively higher intensity in the 1.53 μm emission. These results demonstrate the viability of Er^3+^:YAG nanocrystals as a potential laser material.

## 1. Introduction

In recent years, trivalent erbium (Er^3+^) has been extensively studied for use in a wide variety of applications, including sensing, range finding, medical procedures, and communications [1,2,3], where it is used in near infrared (NIR) lasers. Of particular interest for these applications are eye-safe lasers with a wavelength of 1.53–1.66 μm [1,4,5]. While single-crystal Y_3_Al_5_O_12_ yttrium aluminum garnet (YAG) has long been used as a laser host material doped with Nd^3+^ and other trivalent rare-earth (RE^3+^) ions [6], developments in nanotechnology have drawn a significant amount of attention to transparent ceramic materials [7,8]. In particular, polycrystalline ceramic YAG doped with RE^3+^ ions such as Nd^3+^ and Er^3+^ have been shown to be suitable laser materials, which can be easier and less expensive to grow than their single-crystal counterparts [4,9,10,11,12,13,14].

While the spectroscopic properties of Er^3+^ in single-crystal YAG have been thoroughly studied [15,16], relatively little research has been done with Er^3+^ in polycrystalline ceramic and nanocrystal YAG hosts [17,18,19,20]. Nanocrystals can have the advantage of being faster, less expensive, and easier to grow when compared to both polycrystalline ceramics and single crystals.

In this article, we present the comparative spectroscopic properties of Er^3+^ doped in single-crystal, polycrystalline ceramic, and nanocrystal YAG hosts. Room-temperature fluorescence spectra are taken in the near-infrared region, and the room-temperature fluorescence decay times are measured for the 1650 nm emission and compared between the three samples. Finally, the spectroscopic properties of these materials are analyzed and compared.

## 2. Materials and Methods

A polycrystalline ceramic YAG half-disk of 15.5 mm diameter and 1.56 mm thickness with 50 at.% Er^3+^ content was acquired from Quarles at VLOC [21]. A single-crystal YAG disk of 4 mm diameter and 1.2 mm thickness with 50 at.% Er^3+^ content was obtained from Kokta at Bicron Crystal Products [22]. Details of the production of both samples can be found in prior publications by our group [11,17].

The Er^3+^-doped YAG nanocrystals were prepared through a modified co-precipitation method similar to that outlined by Dai et al. [23]. Samples were prepared according to the stoichiometry of Er_0.06_Y_2.94_Al_5_O_12_ to obtain a dopant concentration of 2.0 at.%. The dopant concentration was selected based on prior work by our group, where similar materials singly-doped with lanthanides exhibited strong emission quenching from non-radiative losses and reabsorption above this concentration [24,25,26,27]. A precursor solution was prepared with stoichiometric amounts of Er, Y, and Al nitrates in 50 mL of deionized water at a total cation concentration of 0.16 M. A 50 mL precipitant solution of 80 mM ammonium bicarbonate (NH_4_HCO_3_) was separately prepared and adjusted to a pH of 10.5 through the addition of ammonium hydroxide (NH_4_OH). Lastly, 0.2 g of ammonium sulfate ((NH_4_)_2_SO_4_) was added to the precipitant solution to aid in dispersion.

The precipitant solution was heated to 40 °C under vigorous stirring, and the precursor solution was added dropwise at 2 mL/min. A white colloidal precipitate of metal hydroxide intermediates was formed, which was then collected through centrifugation and washed through sonication several times with deionized water to remove excess unreacted precursors before being freeze-dried overnight to yield a fine white powder. The dried powder was subsequently calcined at 1100 °C in a tube furnace in a mixed 95% N_2_, 5% H_2_ atmosphere to complete the formation of Er^3+^:YAG nanoparticles, remove absorbed water, and repair defects in the crystal structure to reduce non-radiative energy losses.

## 3. Results

### 3.1. Morphology

**Figure 1:**
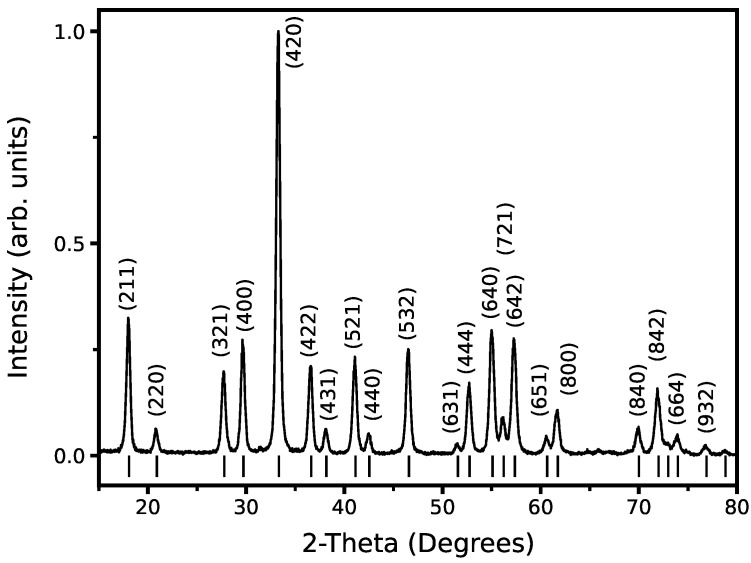
XRD pattern of Er^3+^:YAG nanocrystals with comparison to the reference standard of pure YAG (COD ID: 1529037).

The X-ray diffraction (XRD) pattern (Cu Kα, λ = 1.54 Å) in Figure 1 shows that the Er^3+^:YAG powder matches the YAG reference without any additional phases. The lattice parameter was calculated to be 12.03 ± 0.02 Å, compared to the reference value [28] of 12.01 ± 0.02 Å. The increase in the lattice parameter is attributed to the partial substitution of Y^3+^ by Er^3+^.

Finally, from the Scherrer equation,

(1) $$t=\frac{1.2\lambda }{B\cos\theta }$$

where *t* represents the crystallite size, *B* is the full width at half maximum (FWHM), λ is the X-ray wavelength, and 1.2 is a shape factor used to account for the rectangular shape observed from TEM measurements (JEOL, Tokyo, Japan), the crystallite size for the particles was calculated as 29 nm for the (220) plane, 25 nm for the (420) plane, and 36 nm for the (444) plane. As XRD measurements (Panalytical, Westborough, MA, USA) are taken from powder and thus represent many nanocrystals rather than individual particles, the Scherrer analysis provides an approximation of the lower range of particle sizes rather than being representative of the average particle size.

### 3.2. Microscopy

**Figure 2:**
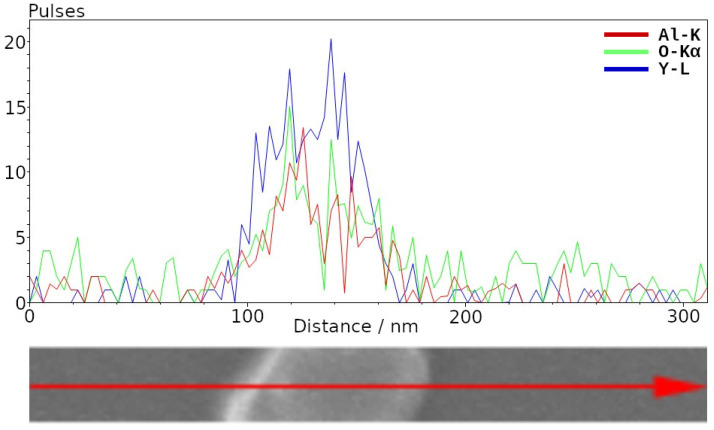
EDS profile of a selected nanoparticle confirming YAG composition. The erbium concentration of 2 at.% is below the sensitivity of the EDS detector.

**Figure 3:**
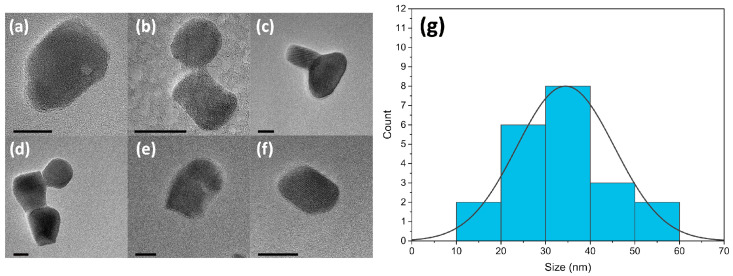
(**a**–**f**) A set of TEM images of Er^3+^:YAG nanoparticles, scale bar in all images is 20 nm. (**g**) Histogram showing particle size distribution profile and average size of the particles.

Elemental analysis was performed using energy-dispersive X-ray spectroscopy (EDS) with a Hitachi S5500 scanning transmission electron microscope (STEM, Hitachi, Tokyo, Japan) equipped with a solid-state Bruker detector (Bruker, Billerica, MA, USA). The results shown in Figure 2 confirm the YAG composition of the nanocrystals. High-resolution transmission electron microscopy (HRTEM, JEOL, Tokyo, Japan) was performed with a JEOL 2010F (JEOL, Tokyo, Japan). TEM images (Figure 3a–e) were used to calculate the size distribution of the synthesized nanocrystals by averaging the horizontal, vertical, and diagonal dimensions, which showed particles ranging from 18 nm to 60 nm with an average particle size of 35 nm, represented by the histogram in Figure 3g. The size distribution observed here provides agreement with the Scherrer calculations of crystallite sizes, which tends to underestimate particle sizes for non-spherical particles.

### 3.3. Spectroscopy

**Figure 4:**
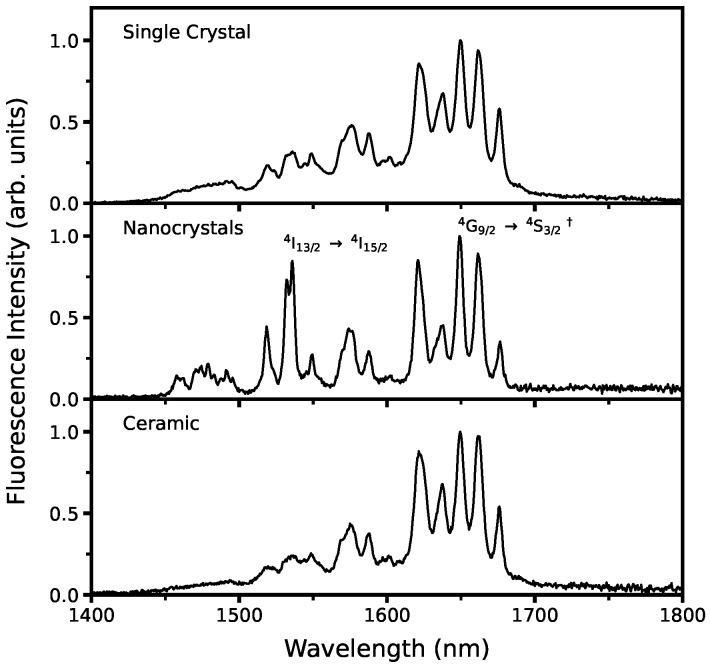
Normalized NIR fluorescence spectra of Er^3+^:YAG single-crystal, nanocrystal, and polycrystalline ceramic at room temperature under 980 nm excitation. ${}^{†}$ Denotes transition used for lifetime calculations.

Figure 4 shows emission spectra in the NIR region, collected at room temperature by exciting the samples with a 980 nm diode laser and collecting the signal with a QuantaMaster 51 spectrofluorometer (PTI, Birmingham, NJ, USA) from Photon Technology International (PTI) equipped with an InGaAs detector. The single-crystal and ceramic samples were excited by directing the laser into the narrow side of the sample and collecting the emission signal from the broad side, to minimize quenching. The nanocrystal powder was placed in a thin quartz cuvette for the measurements. We have previously published a detailed analysis of the energy level transitions of the Er^3+^:YAG single-crystal and polycrystalline ceramic samples used here, where we provide the results of Judd–Ofelt analysis along with measured and calculated fluorescence line strengths and emission cross-sections [11,17].

**Figure 5:**
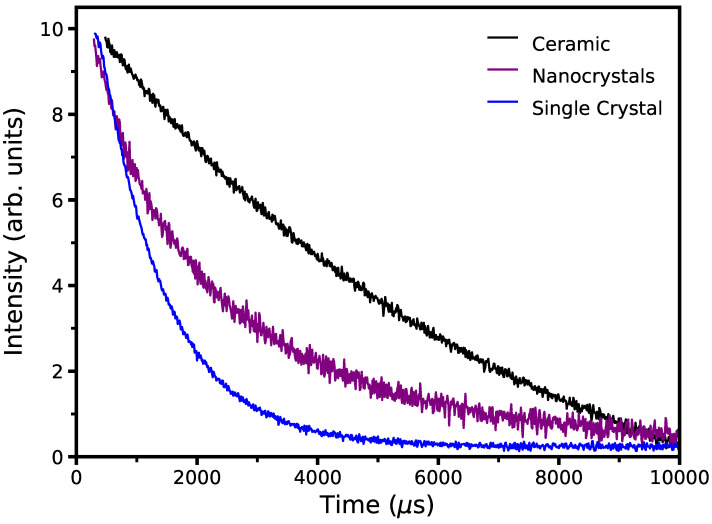
Fluorescence decay times for the 1650 nm emissions of all three samples excited at 525 nm.

Fluorescence decay times shown in Figure 5 for the 1650 nm emission were collected with the PTI system and a nanosecond-pulsed nitrogen pumped dye laser from PTI operated at 525 nm. The 1650 nm emission was chosen for lifetime measurements due to being the most intense emission observed consistently between all three samples. The decay time for each emission was calculated by fitting a single exponential function,

(2) $$I\left(t\right)=A_{1}e^{-t/\tau_{1}}$$

where *A*_1_ represents the fitting parameter and $\tau_{1}$ is the fluorescence decay time. The lifetime of the 1650 nm emission was calculated to be 1.14 ms for the single-crystal, 7.04 ms for the ceramic, and 2.15 ms for the nanocrystals. Due to a tendency for dopants to favor selection sites near the surface of nanoparticles and at grain boundaries [29], the nanocrystals were expected to have a fluorescence lifetime in between that of single-crystals where the dopants are dispersed throughout the crystal lattice and that of polycrystalline ceramics where the dopants tend to aggregate toward the grain boundaries, and this is observed in the measured lifetimes.

### 3.4. Crystal Structure

**Figure 6:**
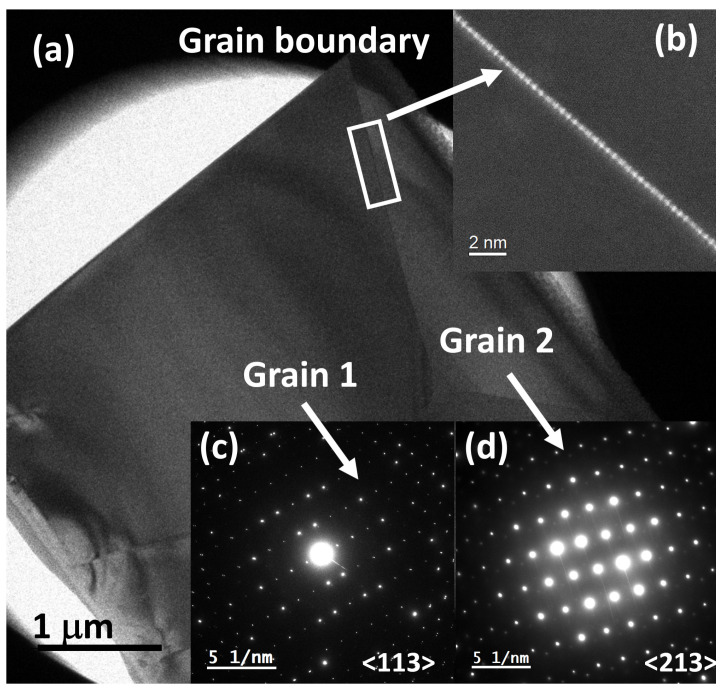
(**a**) Low magnification TEM image of the polycrystalline sample prepared by FIB. (**b**) HAADF-STEM image showing the atomic segregation of erbium atoms at the grain boundary. (**c**,**d**) SAED patterns collected under precession electron diffraction mode with liquid nitrogen cooling.

To perform a study of the crystal grain boundaries, the commercially fabricated 50 at.% Er^3+^:YAG polycrystalline ceramic sample was prepared using a focused ion beam (FIB, Zeiss, Jena, Germany) Zeiss Crossbeam 340 using 30, 5, and 2 kV gallium ions. Selected area electron diffraction patterns (SAED) under precession electron diffraction (PED) mode [30] were collected using a JEOL 2010F TEM operated at 200 kV and equipped with a NANOMEGAS precession unit. PED patterns were obtained under nanobeam diffraction mode with a precession angle of 0.48°, which has been performed to reduce the dynamical effects produced by the multiple interactions within the specimen (Figure 6b,c). In addition, to reduce the thermal diffusion scattering, a Gatan 626 double tilt liquid nitrogen cryo sample holder was used to collect the PED patterns at approximately 96 K. PED patterns were captured using a TVIPS 16 megapixel F416 CMOS camera (TVIPS, Gauting, Germany). Scanning transmission electron microscopy (STEM, JEOL, Tokyo, Japan) was employed to obtain high angle annular dark field (HAADF) images.

HAADF-STEM shows an incoherent image nature that depends on the atomic number (Z) and thickness of the sample (t), where the intensity is approximately [31] I ∝ tZ^2^. In Z-contrast imaging, the diffraction contrast is significantly reduced, resulting in a quantitative chemical-sensitive contrast. With this method, a Z-contrast image was collected at the grain boundary between grains 1 and 2 shown in Figure 6. The spherical aberration corrected (Cs) HAADF-STEM image shows an increase in contrast at the grain boundary due to the migration of erbium atoms (with heavier atomic number Z), see inset of Figure 6b. Chemical profiling was performed using EDS and STEM at the grain boundary, in which an increase in the signal of erbium was measured, as well as a crystallographic phase mapping [30]. The atomic segregation of erbium atoms at the grain boundary is consistent with the migration of atoms at the surface in the nanocrystal counterparts, which results in the optical response reported here.

## 4. Conclusions

The comparison of the NIR emissions is in good agreement between the three hosts, while the nanocrystals demonstrate significantly higher relative emission intensity in the 1.5–1.6 μm emission bands commonly used for medical lasers. As emission quenching occurs through cross-relaxation processes that depend on the distance between dopant ions, the dopant distribution of the nanocrystals presented in this work yields the advantage of a much lower dopant concentration than that required for the bulk materials where dopant ions are distributed throughout the crystal matrix. The fluorescence lifetime of the nanocrystals is within range of the lifetimes measured for the single-crystal and ceramic hosts, indicating comparable internal quantum efficiency between all hosts [32,33]. Electron diffraction and Cs-STEM Z-contrast imaging show clear evidence of erbium segregation at the grain boundaries. This migration of erbium atoms at the interface reduces the optical quantum efficiency compared with the more uniformly-dispersed dopants of the single-crystal. In this regard, the use of Er^3+^:YAG nanocrystals improves the optical response of the polycrystalline nature and ensures they are good candidates for technological applications. Nanocrystal size distribution yielded an average of 35 nm, making the particles suitable for a variety of applications where particle size may be critical. The results of these spectroscopic and morphological studies indicate the viability of Er^3+^:YAG nanocrystals as a synthesis route for the production of transparent ceramics with applications in laser materials.

## Data Availability

Not applicable.

## Abbreviations

The following abbreviations are used in this manuscript:

YAG	Yttrium aluminum garnet
NIR	Near-infrared
RE	Rare-earth
XRD	X-Ray diffraction
FWHM	Full width at half maximum
EDS	Energy-dispersive X-ray spectroscopy
STEM	Scanning transmission electron microscope
TEM	Transmission electron microscopy
HRTEM	High-resolution transmission electron microscopy
PTI	Photon technology international
FIB	Focused ion beam
SAED	Selected area electron diffraction
PED	Precession electron diffraction
HAADF	High-angle annular dark field
Cs	Spherical aberration correction
